# Reverse ADOR: reconstruction of UTL zeolite from layered IPC-1P†

**DOI:** 10.1039/d1ma00212k

**Published:** 2021-04-02

**Authors:** Ondřej Veselý, Pavla Eliášová, Russell E. Morris, Jiří Čejka

**Affiliations:** Department of Physical and Macromolecular Chemistry & Charles University Center of Advanced Materials, Faculty of Science, Charles University Hlavova 8 12843 Prague Czech Republic jiri.cejka@natur.cuni.cz; School of Chemistry, EaStChem, University of St. Andrews North Haugh St. Andrews Fife KY16 9ST UK

## Abstract

The assembly–disassembly–organisation–reassembly (ADOR) process has led to the discovery of numerous zeolite structures, albeit limited to materials with decreased pore size in relation to the parent germanosilicate zeolite. This limitation stems from the rapid decrease in *d*-spacing upon hydrolysis (disassembly). Nevertheless, we have artificially increased the *d*-spacing of layered IPC-1P by intercalating organic species. Furthermore, we have reconstructed double four rings (D4R) between layers, thus transforming IPC-1P back into the parent **UTL** zeolite. This reconstruction has provided not only germanosilicate but also a new, high-silica **UTL** zeolite (Si/Ge = 481). Therefore, our “reverse ADOR” opens up new synthetic routes towards promising extra-large-pore zeolite-based materials with new chemical compositions.

## Abstract 2

The germanosilicate zeolite UTL was reconstructed from the layered precursor IPC-1P using the modified Assembly–Disassembly–Organisation–Reassembly (ADOR) process. The reverse ADOR is a promising new route for synthesis of extra-large-pore zeolites.

## Graphical abstract

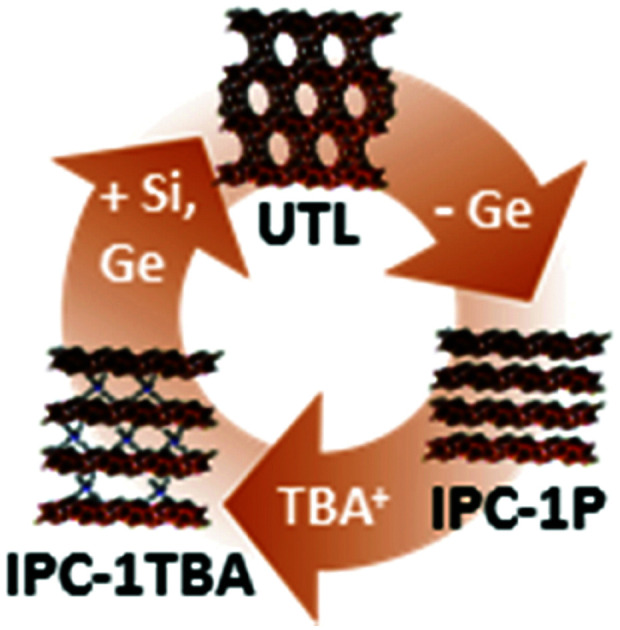

## 1. Introduction

Zeolites are crystalline silicate-based microporous materials. The micropore size of common zeolites is similar to the kinetic diameters of small organic molecules.^1^ For this reason, zeolites are extensively used in separation and shape-selective catalysis processes. For example, they are commonly applied as heterogeneous catalysts in petrochemistry.^2–4^ Moreover, zeolites have also been modified to catalyse biomass conversion^5–7^ and fine chemical synthesis,^8,9^ highlighting the wide range of industrial uses of these materials.

Zeolites are commonly prepared by solvothermal (mostly hydrothermal) synthesis^10^ in the presence of structure directing agents (SDAs) and mineralising agents (OH^−^ or F^−^).^11^ While the hydrothermal method is versatile and easy to perform, its mechanism remains difficult to generalise. As a result, new zeolites are often discovered by trial and error. In contrast, the assembly–disassembly–organisation–reassembly (ADOR) method has been developed to prepare new zeolites and to predict their structure based on theoretical calculations and experimental conditions.^12,13^ The ADOR process exploits labile Ge-rich double-four ring (D4R) units in germanosilicates, such as **UTL** or *CTH.^14,15^ The structure of these zeolites consists of Si-rich layers connected by Ge-rich D4Rs. Upon selective hydrolysis of D4Rs, layered materials are formed, thus preserving their original structure. Subsequently, these layers undergo topotactic condensation to a new 3D structure and hence a new zeolite. Accordingly, the ADOR is a tool for the rational design of new zeolites by controlled 3D-2D-3D transformation.^14^

**Fig. 1:**
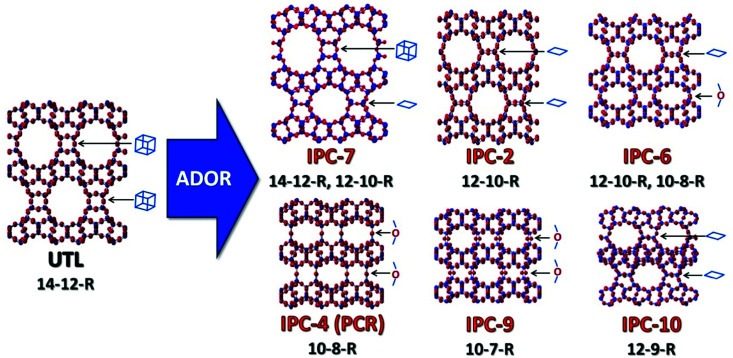
Zeolite UTL and its daughter structures prepared using the ADOR method, highlighting the connecting units between the layers in the different materials.

The ADOR process was first studied on the **UTL** germanosilicate, which hydrolyses to layered IPC-1P.^16^ Further manipulations of IPC-1P layers have resulted in a whole new family of materials (**PCR**, **OKO**, *PCS, IPC-7, IPC-9 and IPC-10; Fig. 1).^17–20^ The IPC materials contain the same Si-rich layers as the parent **UTL** but differ in the connections between layers and consequently in pore size. The average pore size of IPC materials, 12-ring and 10-ring, is usually smaller than that of the parent structure (14-ring). However, no **UTL** or other structure with 14-ring pores has been formed after full 3D-2D-3D transformation until now.

The kinetics of **UTL** hydrolysis has been described in detail by Henkelis *et al.*^21^**UTL** rapidly disassembles into IPC-1P, thereby decreasing the interlayer distance (as measured by the *d*_200_-spacing in XRD) from 1.45 nm to 1.05 nm. As the IPC-1P layers slowly rearrange, an IPC-2P with 1.18 nm *d*-spacing is formed, but this IPC-2P is never fully reconverted into **UTL** under such conditions.^22^ Conversely, Xu *et al.* succeeded in doing so when using another Ge-rich **UTL**, which was transformed back to **UTL** through isomorphous substitution of Ge by Si. This Ge-rich **UTL**, however, still contained the SDA. The SDA molecules were necessary to preserve the structure throughout the process, otherwise **UTL** would quickly disassemble into layered IPC-1P. Once the layered precursor was formed, the UTL structure could not be restored,^23^ so **UTL** restoration from layered IPC-1P remains a challenge. Wu *et al.* have also reported a 2D–3D transformation of the layered material MCM-22P.^24,25^ After expanding the interlayer distance by intercalating organic agents, they connected the expanded layers through silanes, thus forming MCM-IEZ. These authors used a similar approach to transform the layered HUS-2 to ECNU-19. However, in both MCM-IEZ and ECNU-19, the layers were not connected by D4R units, which are present in **UTL**, but instead by individual Si linkages. Therefore, by definition, these materials are not zeolites.^26^

Considering the above, in this study we have developed a method for reversing the ADOR process and fully restoring the **UTL** zeolite from layered IPC-1P for the first time. In this approach, **UTL** reconstruction relies on the formation of D4R units between layers. D4Rs form either in a favourable ratio of Si and Ge or in the presence of additional agents, such as fluorides. The process results in a **UTL** zeolite with Si/Ge ratios ranging from 8 to 400, depending on synthesis design.

## 2. Experimental section

### 2.1 Synthesis of the structure-directing agent (SDA) for **UTL**

(6*R*,10*S*)-6,10-Dimethyl-5-anizosporo[4.5]decane hydroxide was used as the SDA to synthetize the UTL germanosilicate.^27^ In total, 60 mL of 1,4-dibromobutane, 82.9 g of K_2_CO_3_ and 500 mL of acetonitrile were mixed in a round-bottom flask. Subsequently, 67 mL of 2,6-dimethylpiperidine was added dropwise, and the mixture was heated to 85 °C and kept refluxing overnight. The acetonitrile was evaporated, and the solid product was dissolved in ethanol. Insoluble compounds were filtered off. Ethanol was evaporated to create an almost saturated solution. Then, the product was precipitated by adding diethyl ether, filtered off, washed with ether, and dried under vacuum overnight. The identity of the SDA structure was confirmed by ^1^H NMR spectroscopy. The product was ion exchanged to the hydroxide form using the Ambersep 900(OH) ion exchange resin.

### 2.2 **UTL** synthesis

**UTL** was prepared using the hydrothermal method.^28^ Germanium dioxide was dissolved in SDA solution in water. Then, silica (Cab–O–Sil M5) was added to the solution, and the mixture was stirred at room temperature until completely dissolved. The resulting mixture, with a molar composition of 0.67SiO_2_:0.33GeO_2_:0.4SDA:33.3H_2_O, was charged into a 1000 mL Parr autoclave and heated to 175 °C for 6 days with agitation (200 rpm). The solid product was recovered by filtration, washed out with a copious amount of distilled water and dried in the oven at 60 °C. The SDA was removed by calcination in air at 550 °C for 6 h, with a temperature ramp of 1 °C min^−1^.

### 2.3 IPC-1P synthesis

Calcined **UTL** zeolite was hydrolysed in 1 M CH_3_COOH (250 mL per g of sample) at 85 °C for 16 h. The product was isolated by filtration, washed with water and dried at 60 °C.

### 2.4 Intercalation

IPC-1P was treated with a 20% solution of tetrabutylammonium hydroxide (TBAOH) for 6 hours at room temperature.^27^ The product was recovered by centrifugation and washed with distilled water to neutral pH. The intercalated precursor (IPC-1TBA) was dried at 60 °C overnight.

### 2.5 D4R restoration

In total, 0.1 g of IPC-1TBA was added to a 25 mL Teflon-lined steel autoclave with 5 mL of 1.25 M HCl in ethanol and the respective sources of silicon and germanium (see Table 1). The autoclave was heated to 170 °C for 20 hours. The product was filtered off, washed with ethanol and dried at 60 °C. The TBA was removed by calcination in air at 550 °C for 6 h, with a temperature ramp of 1 °C min^−1^.

**Table 1:** Molar ratios of the Si and Ge sources in the individual reaction mixtures and their respective labelling

Sample	(EtO)_2_Me_2_Si	(EtO)_2_Me_4_Si_2_O	Me_8_Si_4_O_4_	POSS	(MeO)_4_Ge
rec Si1	65.8 mg	—	—	—	—
rec Si2	—	49.4 mg	—	—	—
rec Si4	—	—	26.7 mg	—	—
rec Si8	—	—	—	29.8 mg	—
rec Si–Ge (3 : 1)	49.4 mg	—	—	—	21.9 mg
rec Si–Ge (1 : 1)	32.9 mg	—	—	—	43.7 mg
rec Si–Ge (1 : 3)	16.5 mg	—	—	—	65.6 mg
rec Ge	—	—	—	—	87.3 mg

### 2.6 Characterisation

The crystalline structure of the samples was determined by X-ray powder diffraction (XRD) on a Bruker AXS D8 Advance diffractometer with a Vantec-1 detector in the Bragg–Brentano geometry using Cu Kα radiation (1.54056 Å).

High-resolution transmission electron microscopy (HRTEM) images and energy dispersive X-ray (EDX) spectra were acquired under a JEOL NEOARM 200 F microscope with a Schottky-type field emission gun at an accelerating voltage of 200 kV. The samples were dispersed onto the carbon-coated copper grids before the measurements.

Ar adsorption/desorption isotherms were collected at −186 °C on a 3Flex (Micromeritics) static volumetric apparatus. All samples were degassed on a SmartVac Prep (Micromeretics) at 300 °C under vacuum for 8 h before the sorption measurements. The surface area was calculated using the BET method and adsorption data on a relative pressure range of *p*/*p*°_0_ = 0.05–0.25. The *t*-plot method was applied to determine the micropore volume (*V*_mic_). The adsorbed amount at a relative pressure of *p*/*p*°_0_ = 0.975 reflects the total micropore volume and interparticle adsorption (*V*_tot_). The pore size distributions were calculated using the Horwath–Kawazoe method.

## 3. Results and discussion

### 3.1 Intercalation

**Fig. 2:**
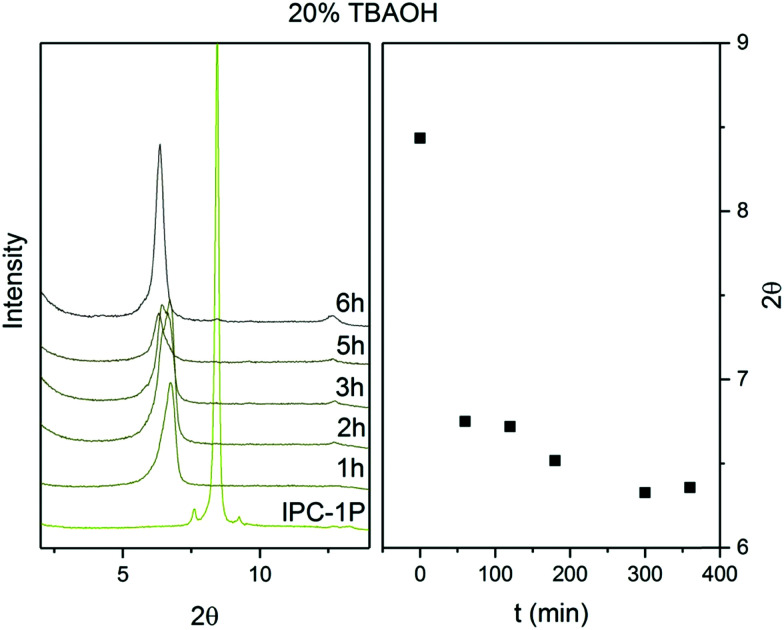
Powder XRD patterns and positions of 200 diffraction lines of IPC-1P intercalated with TBA^+^ over time.

The *d*-spacing between the respective layers in the **UTL** structure is 1.44 nm (which corresponds to reflection at 6.14° in X-ray diffraction using Cu Kα radiation). Hydrolysis of the D4R units in **UTL** results in the layered material IPC-1P. IPC-1P consists of silica-rich layers with a *d*-spacing of 1.05 nm (corresponding to reflection at 8.41°). The first challenge in reconverting IPC-1P into **UTL** was to increase the spacing between IPC-1P layers to 1.44 nm; however, the distance between IPC-1P layers cannot spontaneously increase to the original value (ref. 21; Fig. S1, ESI†). Hence, we adjusted this distance by intercalation. Intercalation, or swelling, of layered silicates and zeolites commonly involves surfactants, such as cetyltrimethylammonium hydroxide.^29–31^ However, their long hydrocarbon chains are flexible, and their structure between the layers is sensitive to the pH of the environment. Because this may produce disorder and irregularities in the reconstructed material,^32^ we decided to intercalate the layers with more rigid species, such as the tetrabutylammonium cation (TBA^+^).^27^ TBA^+^ intercalation into IPC-1P shifted the interlayer 200 reflection from XRD from 8.4° to 6.37°, which is relatively close to 6.14° (Fig. 2) – the position of the 200 reflection of the UTL material.^33^ Accordingly, TBA^+^ is suitable for increasing the *d*-spacing of IPC-1P to a distance close to that of the **UTL** zeolite.

To optimise the intercalation time, we performed a kinetic experiment with a 20% TBAOH solution. This solution has a basic pH, which is necessary to deprotonate the IPC-1P layers, to form silanolates, and to break the H-bonds between the layers.^34^ However, high pH also causes desilication,^35–38^ creating defects and mesopores in the material, which loses layer crystallinity. To preserve the crystallinity of the IPC-1P layers, we shortened the intercalation time. Fig. 2 shows the stabilisation of the 200 reflection at 6.37° for 6 hours. After 6 hours, the 200 peak position remains constant under the conditions of the treatment, thus indicating that the intercalation has ended.

### 3.2 Reconstruction

**Fig. 3:**
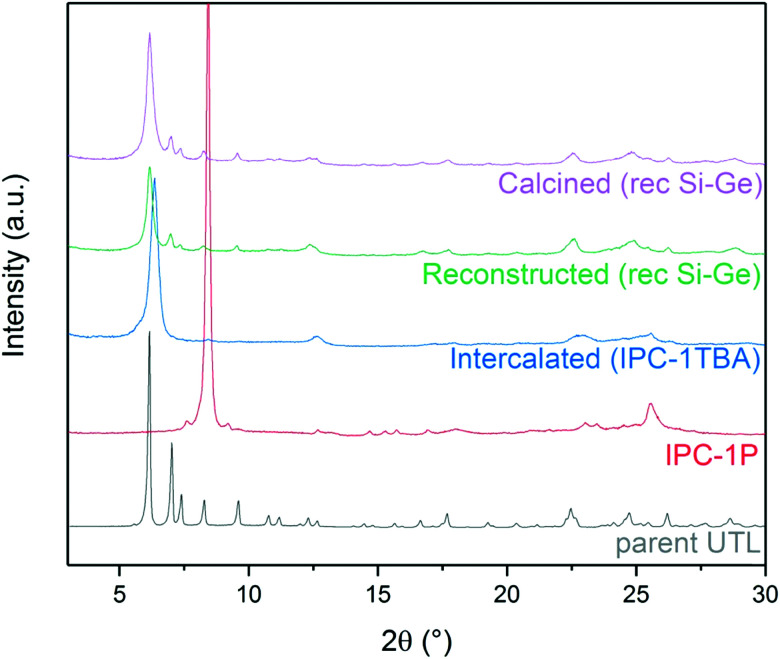
Powder XRD patterns of individual stages in the IPC-1P-to-**UTL** reconstruction, using the rec Si–Ge (1 : 1) sample as an example.

The aim, and the main challenge, of this study was to restore the **UTL** structure by reconstructing D4R units between IPC-1TBA layers. Numerous D4R-containing zeolites, including UTL, crystallise mainly as germanosilicates; therefore, we reconstructed UTL using silicon and germanium alkoxides and their combination for their good reactivity and solubility (see Section 3.3). After silicon and germanium incorporation, the 200 diffraction shifted from 6.37° to 6.16° (Fig. 3). New diffraction lines also appeared at 6.99, 7.35, 8.27, 9.55, 16.7 and 17.7°. These diffraction lines match the XRD pattern of the **UTL** structure. Moreover, these reflections remained unchanged after calcining the sample to remove the TBA^+^. These findings suggest that the reconstructed **UTL** is stable without the support of organic agents.

**Fig. 4:**
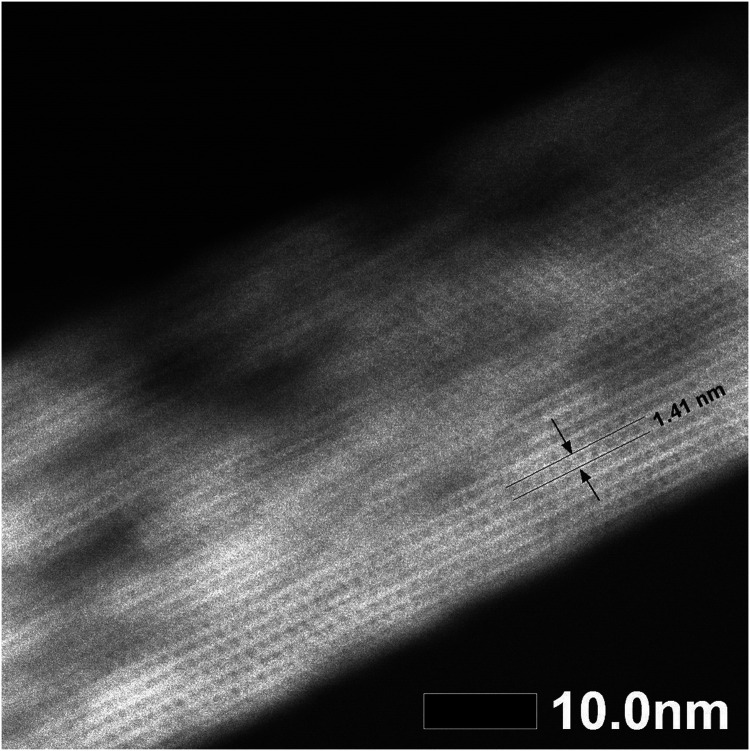
STEM image of the reconstructed **UTL** sample.

The STEM image (Fig. 4) confirmed that the distance between the layers is 1.41 nm and that the layers are visibly connected as a three-dimensional framework. However, STEM also revealed that the treatments caused some etching of the crystal, thereby forming mesopores in the zeolite. The formation of these mesopores is further supported by the changes in textural properties outlined in Table 2.

**Table 2:** Textural properties of the parent **UTL** and reconstructed **UTL** material determined by Ar adsorption

	BET (m^2^ g^−1^)	*S*_ext_ (m^2^ g^−1^)	*V*_tot_ (cm^3^ g^−1^)	*V*_mic_ (cm^3^ g^−1^)	Si/Ge
Parent **UTL**	620	52	0.27	0.25	4.47
Reconstructed **UTL**	808	188	0.48	0.15	7.50

### 3.3 Ge content effect

Germanium can stabilise D4R units in zeolites.^39,40^ The results shown above illustrate the structure directing effect of germanium on **UTL** reconstruction. We investigated how the germanium content affects the result in a series of similar reconstruction experiments with varying Si : Ge molar ratios (we use the “Si : Ge” notation to express the ratio of Si and Ge sources that we added to the reconstruction mixture to differentiate this ratio from the overall Si/Ge ratio of bulk samples).

**Fig. 5:**
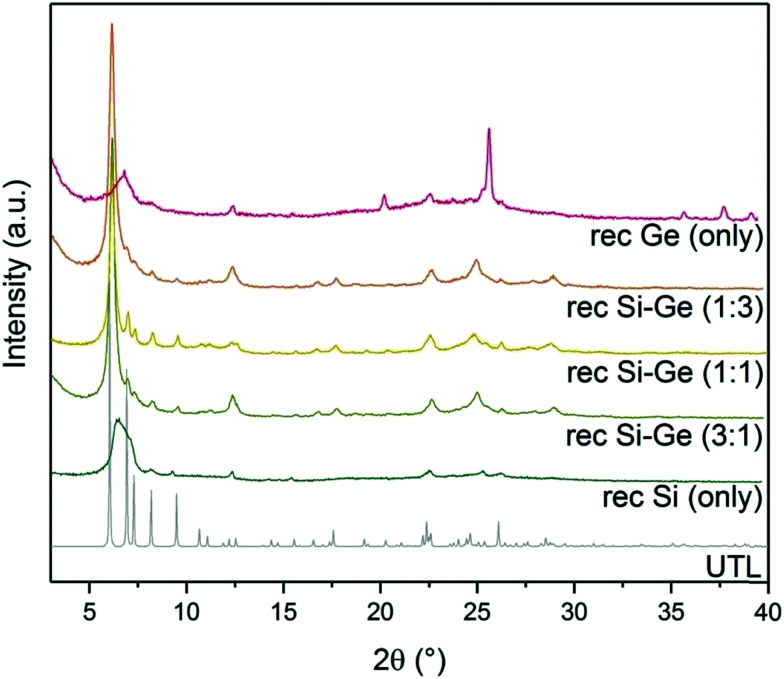
Powder XRD patterns of calcined samples after reconstruction with different Si:Ge compositions.

XRD showed a broad diffraction peak at 7° (Fig. 5) in the sample reconstructed without germanium. The broad peak is caused by a non-uniform interlayer distance, thus implying that the layers lost their ideal ordering upon calcination. The loss of ordering likely stems from unsuccessful or incomplete reconstruction of the D4R units that connect individual layers. The samples reconstructed with both Si and Ge resulted in **UTL** structures. The peaks in XRD are less pronounced in the samples with 1 : 3 and 3 : 1 Si : Ge, mainly in the region from 5 to 10 °C. These patterns suggest that the interlayer ordering contains some defects. The sample with 1 : 1 Si : Ge produced the powder XRD pattern closest to that of the parent UTL zeolite. When we used only germanium for the reconstruction, the structure also collapsed upon calcination. Moreover, the diffraction pattern also contained new peaks at 25.7, 35.7, 37.7 and 39.2° belonging to germanium oxide. The germanium oxide species also appeared in the STEM image of the sample (Fig. S2, ESI†), which had a very low micropore volume (0.07 cm^3^ g^−1^; Table 3). The formation of germanium oxide may result from the high reactivity of germanium methoxide, which forms the oxide before it can be incorporated into the framework. Alternatively, the purely Ge-based D4Rs may be unstable under the current conditions, but this assumption requires further investigation, which is beyond the scope of this article.

**Table 3:** Textural properties of samples after reconstruction with different Si:Ge compositions determined by Ar adsorption

	BET (m^2^ g^−1^)	*S*_ext_ (m^2^ g^−1^)	*V*_tot_ (cm^3^ g^−1^)	*V*_mic_ (cm^3^ g^−1^)
**UTL**	620	52	0.27	0.25
rec Si (only)	645	334	0.48	0.12
rec Si–Ge (3 : 1)	550	156	0.35	0.12
rec Si–Ge (1 : 1)	808	188	0.48	0.15
rec Si–Ge (1 : 3)	555	165	0.36	0.11
rec Ge (only)	344	139	0.34	0.07

**Fig. 6:**
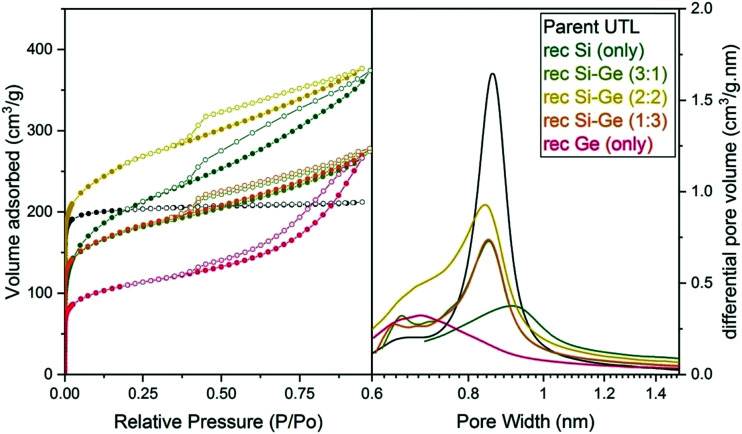
Argon adsorption–desorption isotherms (left) and H–K micropore size distributions (right) of samples after reconstruction with different Si:Ge compositions.

The micropore volume of the reconstructed **UTL** samples ranges from 0.11 to 0.15 cm^3^ g^−1^ (Fig. 6) and is thus smaller than the micropore volume of the parent **UTL** (0.25 cm^3^ g^−1^). These poor textural properties may be caused by incomplete D4R reconstruction because intercalated TBA^+^ occupy some D4R positions. Consequently, D4R reconstruction does not proceed until the intercalant is removed. To test this hypothesis, we performed D4R reconstruction with 1 : 1 Si : Ge, as described above, calcined the sample and repeated the reconstruction under the same conditions. However, we observed only negligible differences in powder XRD patterns (Fig. S3, ESI†) and adsorption behaviour (Fig. S4, ESI†). Nevertheless, the pore volume of the samples decreased after the second reconstruction cycle (Table S1, ESI†) due to further damage under such harsh conditions. This experiment disproved that TBA^+^ significantly hinders D4R reconstruction.

**Table 4:** Textural properties of the samples after reconstruction with different Si:Ge compositions determined by Ar adsorption, with NH_4_F

	BET (m^2^ g)	*S*_ext_ (m^2^ g^−1^)	*V*_tot_ (cm^3^ g^−1^)	*V*_mic_ (cm^3^ g^−1^)
**UTL**	620	52	0.27	0.25
Pure Si	691	341	0.56	0.05
Si : Ge 1 : 1	555	281	0.46	0.06
Pure Ge	280	101	0.26	0.07

Considering the presence of germanium in its D4R, we theorised that the reconstructed **UTL** may be able to undergo hydrolysis to IPC-1P. To test this hypothesis, we performed a hydrolysis of the reconstructed sample in water and in solution of acetic acid at 85 °C. In both cases the 200 reflection on the XRD pattern shifted to higher angles (Fig. S5, ESI†). The shift suggests that the material transformed during the hydrolysis. However, neither of the experiments produced the IPC-1P, probably due to low germanium content or uneven germanium distribution which prevented complete disassembly into layers.

### 3.4 Stabilisation by fluorides

Fluoride anions can also stabilise D4R units in zeolites. In fact, numerous D4R-containing extra-large-pore zeolites have been prepared in fluoride medium.^39,41,42^ Based on the above, we assessed the effect of fluorides on D4R reconstruction. We performed another set of experiments with varying Si:Ge contents, with and without ammonium fluoride.

**Fig. 7:**
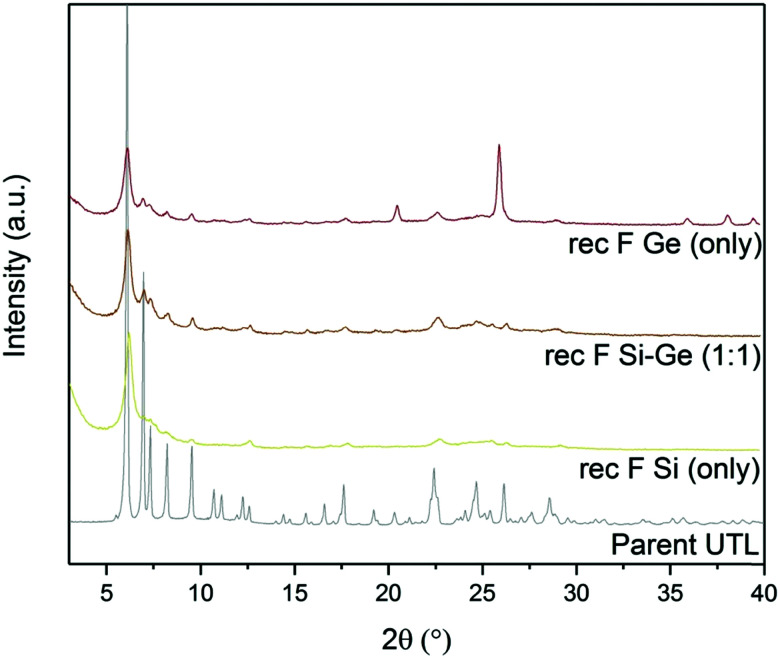
Powder XRD patterns of calcined samples after D4R reconstruction with different Si:Ge compositions, with and without NH_4_F.

The powder XRD patterns (Fig. 7) of samples reconstructed with Si or Ge alone changed significantly after adding ammonium fluoride. The samples that were synthesized with fluoride showed 200 diffraction at 6.16°, similar to **UTL**. This diffraction remained unchanged even after calcination. Other reflections, characteristic of the **UTL**, also appeared at 6.99, 7.35, 8.27 and 9.55° in the sample reconstructed solely with Ge in fluoride-containing medium. However, the powder diffraction pattern of this sample also contained the peaks of germanium oxide. The sample reconstructed with Si alone showed a similar change after adding fluorides, but the other diffraction lines were significantly less intense. The XRD pattern of the sample prepared with 1 : 1 Si : Ge shows no significant difference after adding ammonium fluoride.

**Fig. 8:**
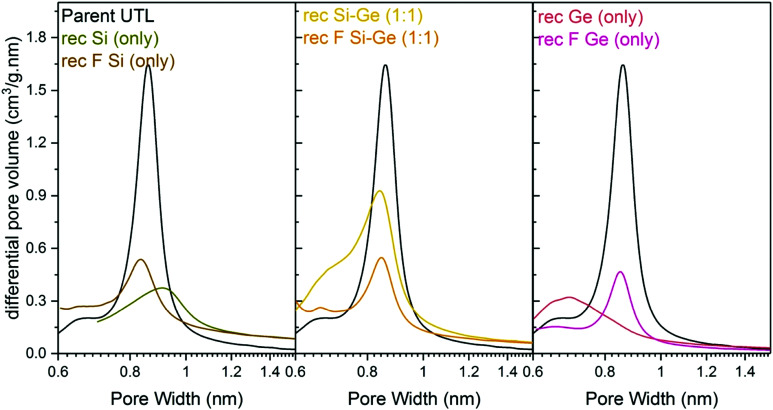
H–K micropore size distributions of samples after reconstruction with different Si:Ge compositions, with and without NH_4_F.

Structural changes caused by fluoride addition to the mixture are also identified in the pore-size distribution. The shape of the distribution curves (Fig. 8) of Ge- and Si-only samples narrowed down. Simultaneously, the maxima of the distribution shifted to 0.84 nm, near the 0.85 nm of the parent UTL. However, all samples prepared in fluoride media had micropore volumes smaller than 0.07 cm^3^ g^−1^ (Table 4) far lower than that of the parent UTL (0.25 cm^3^ g^−1^; Table 2). This decrease may originate from framework etching by fluoride anions in solution, leading to partial amorphisation of the material (Fig. S7, ESI†) and/or subsequent pore blockage by amorphous framework debris.

In summary, while fluoride anions stabilise D4R units, they simultaneously damage the material, which accounts for the poor micropore volumes of the samples.

### 3.5 Structure of the silicon source

**Fig. 9:**
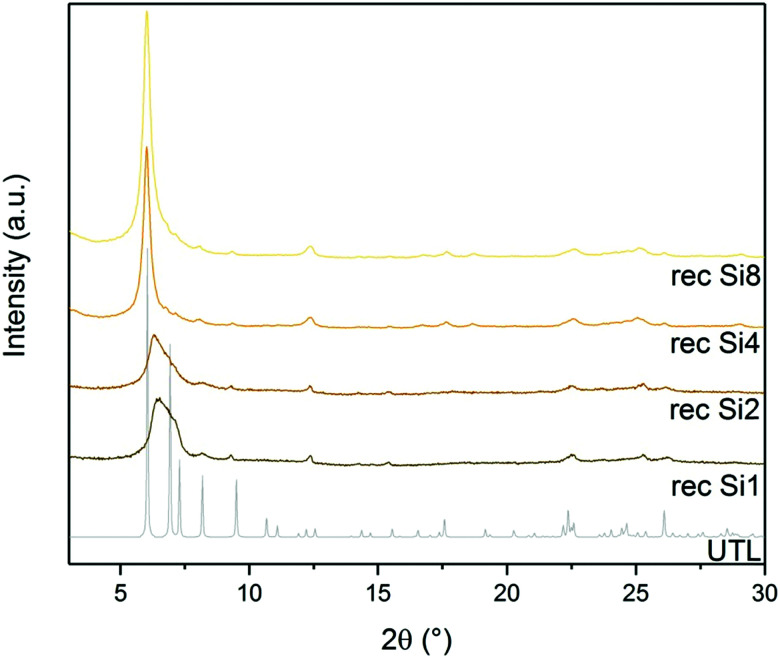
Powder XRD patterns of calcined samples after D4R reconstruction with different Si sources.

D4R formation solely from silane molecules is unfavourable without fluoride anions to stabilise these units. For this reason, we investigated whether other Si sources would form D4R units, even without using germanium or fluorides, *e.g.*, diethoxydimethylsilane (Si1), 1,3-diethoxy-1,1,3,3-tetramethyl-disiloxane (Si2), 2,4,6,8-tetramethylcyclotetrasiloxane (Si4) and octamethylsilsesquioxane (Si8) as Si sources. The broad diffraction peak at 7° in both Si1 and Si2 samples (Fig. 9) implies that their relative layer arrangements lack order and that their structure collapsed after calcination. In contrast, samples Si4 and Si8 retained a very narrow 200 diffraction peak at 6.16°, even after the calcination. The other diffractions at 6.99, 7.35, 8.27 and 9.55° remained unchanged but were less intense than in the germanium-containing sample, as shown in Fig. 3. This lower intensity may arise from the higher silicon content of the samples Si4 and Si8.

**Fig. 10:**
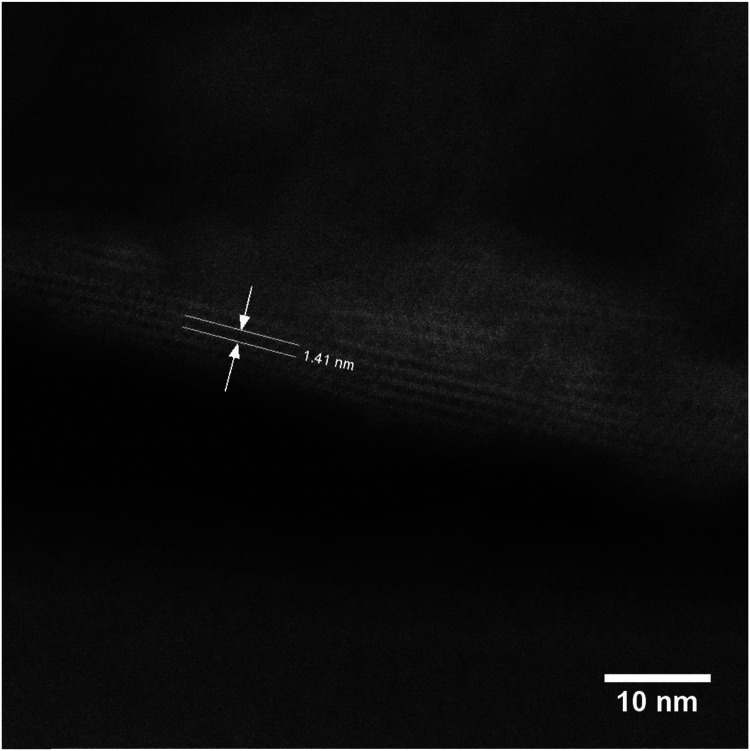
STEM image of the reconstructed **UTL** sample Si4.

STEM measurements (Fig. 10) further confirmed the structure and interlayer spacing, showing 1.41 nm distances between individual layers. This distance is in line with the **UTL** structure. We analysed the composition of the sample Si4 by EDX. The sample has a Si/Ge ratio of 481. This is an interesting result because D4R-containing zeolites or any extra-large pore zeolite with such a high Si content are seldom prepared without any hetero-element (such as Ge) or fluorides.^43^

### 3.6 Tuning the textural properties

**Fig. 11:**
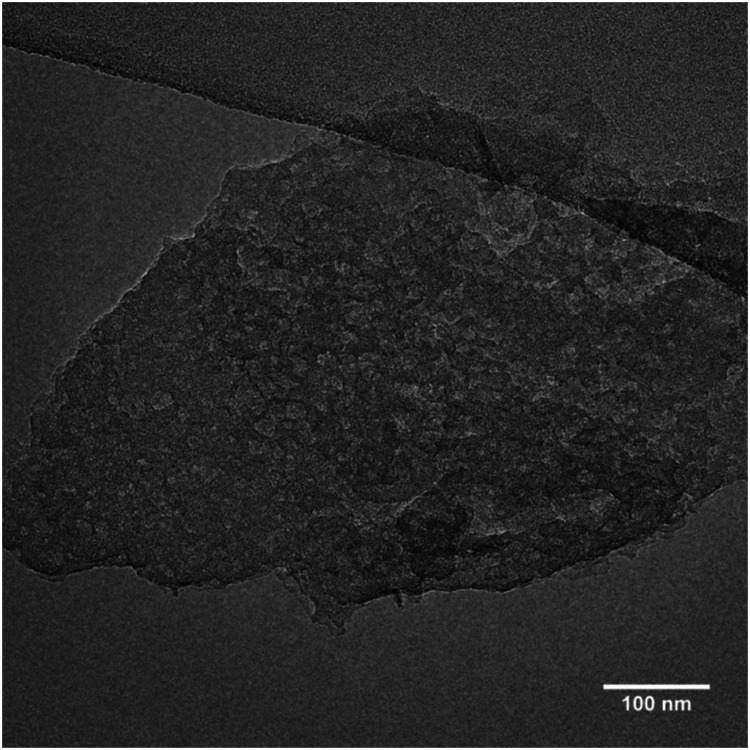
TEM image of the damaged IPC-1TBA sample crystal.

Regardless of their elemental composition, all the **UTL** samples that were reconstructed in this study had lower micropore volumes, ranging from 0.11 to 0.15 cm^3^ g^−1^, than their parent **UTL** sample (0.25 cm^3^ g^−1^). Their lower pore volumes stem from their decreased crystallinity inflicted during the intercalation of the samples with TBA^+^. The IPC-1TBA crystal shown in Fig. 11 is severely damaged after intercalation because the treatment resulted in formation of defects and mesopores in the crystal. However, this is not necessarily a disadvantage of the method as the formation of defects may be beneficial because mesopores can enhance diffusion in catalytic applications.^44^

**Fig. 12:**
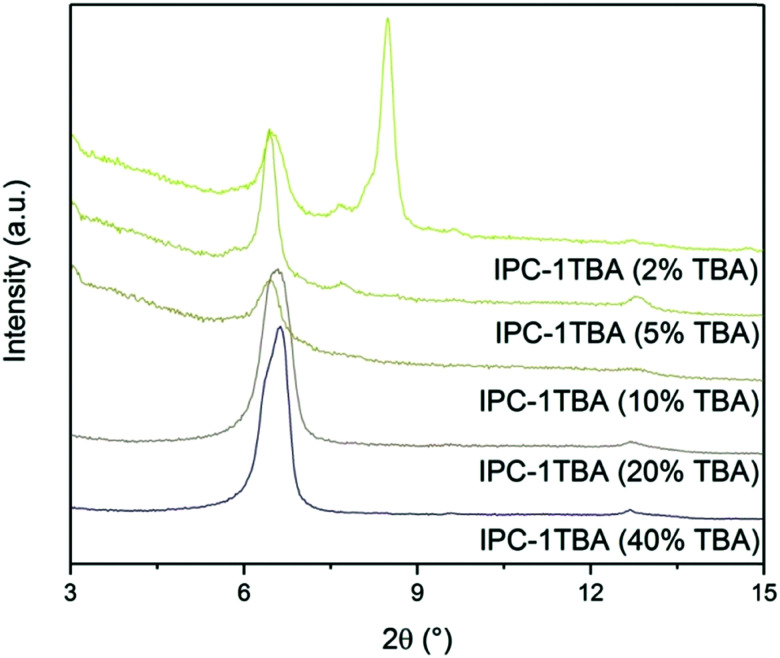
Powder XRD patterns of IPC-1P samples intercalated with TBAOH of varying concentrations.

The high pH of the treatment solution causes etching but is needed for TBA^+^ intercalation. Under optimal conditions, intercalation would proceed at the lowest pH possible to minimise the damage to the crystals, albeit high enough to support TBA^+^ intercalation. To find the optimal conditions, we intercalated IPC-1P with 40, 20, 10, 5 and 2% TBAOH solutions and monitored the position of the 200 diffraction (Fig. 12). The position of 200 peak in the samples treated with 40, 20 and 10% TBAOH was identified at 6.37°. The powder XRD pattern of the sample treated with 5% TBAOH also contained a peak at 6.37° but another, minor diffraction appeared at 7.67°. Treatment with 2% TBAOH resulted in three diffractions at 6.37, 7.67 and 8.4°, indicating incomplete intercalation. In summary, 5% is the minimum TBAOH concentration required for successful TBA^+^ intercalation into IPC-1P.

**Fig. 13:**
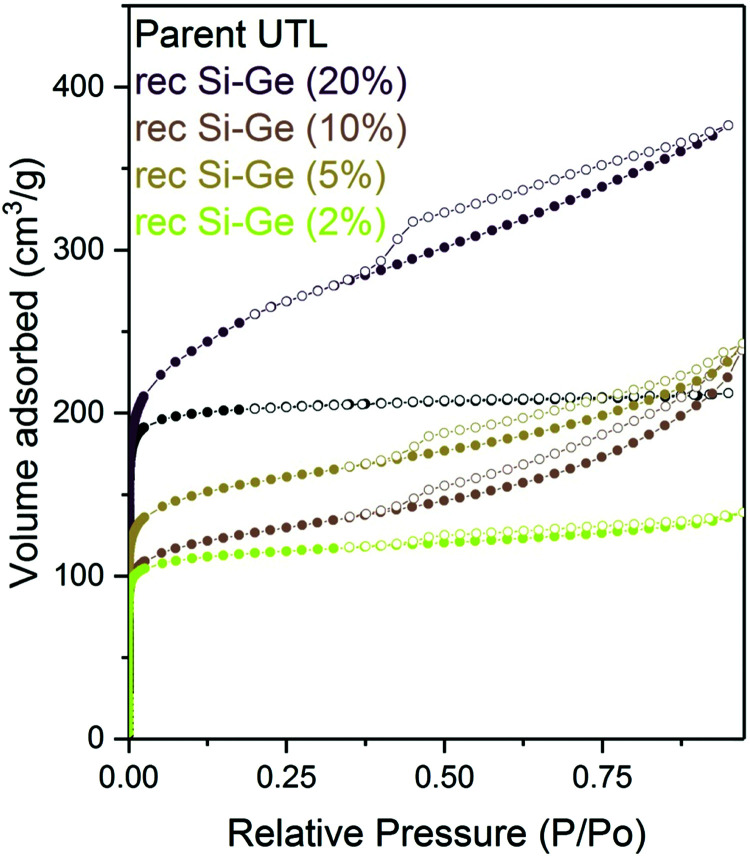
Argon adsorption–desorption isotherms of reconstructed samples previously intercalated with TBAOH of varying concentrations.

We reconstructed all samples with 1 : 1 Si : Ge, including the sample treated with a 2% TBAOH, and subsequently characterised them by adsorption. All isotherms showed large adsorbed amounts at a relative pressure bellow 0.1 (Fig. 13) – filling of micropores. At higher pressures, the intake decreased in all samples; however, the flat plateau observed in the parent **UTL** sample was not found, suggesting that the samples contain not only micropore but also some mesopores. Nevertheless, the slope of the isotherm above the relative pressure *p*/*p*_0_ of 0.1 decreases with the decrease in the concentration of the TBAOH solution. The lower hydroxide concentration mitigates the damage and produces samples with fewer defects.

## 4. Conclusions

Until now, the ADOR method was limited to decreasing the interlayer distance and, consequently, the pore size of the parent zeolite during the process. In this study, we overcome this limitation by intercalating organic species between IPC-1P layers formed by UTL hydrolysis. TBAOH intercalation increases the *d*-spacing to 1.41 nm, the distance of the original UTL structure. The UTL structure is then restored using varying Si and Ge ratios to rebuild the D4R units between the intercalated IPC-1P layers, and the formation of D4Rs is promoted in the presence of an optimal Ge content or fluoride ions. Moreover, the UTL zeolite can also be restored using structurally more complex compounds, such as cyclotetrasiloxanes or polyhedral silsesquioxanes. In summary, the “Reverse ADOR” produces zeolites with a wide range of various compositions, ranging from the common UTL with a 7.5 Si/Ge to the new high-silica UTL with 481 Si/Ge.

While the intercalation treatment inherently causes the formation of mesopores in the zeolite crystals, we can mitigate its impact by tuning the treatment conditions. Furthermore, these mesopores may, in turn, be advantageous in future catalytic applications. Therefore, the “Reverse ADOR” method opens up opportunities for incorporating other elements towards producing novel zeolite-based catalysts.

## Conflicts of interest

There are no conflicts to declare.

## Supplementary Material

MA-002-D1MA00212K-s001
