# Supplementary material for: Reverse ADOR: reconstruction of UTL zeolite from layered IPC-1P
Source: Mater Adv. 2021 Apr 2;2(12):3862–70. doi: 10.1039/d1ma00212k (PMC8216593; doi:10.1039/d1ma00212k)
Supplement: MA-002-D1MA00212K-s001 [file MA-002-D1MA00212K-s001.pdf]

Electronic Supplementary Material (ESI) for Materials Advances.
This journal is © The Royal Society of Chemistry 2021

Supporting Information

Reverse ADOR: Reconstruction of UTL zeolite from layered IPC-1P

Ondřej Veselý[1], Pavla Eliášová[1], Russell E. Morris[2]★ and Jiří Čejka[1]★

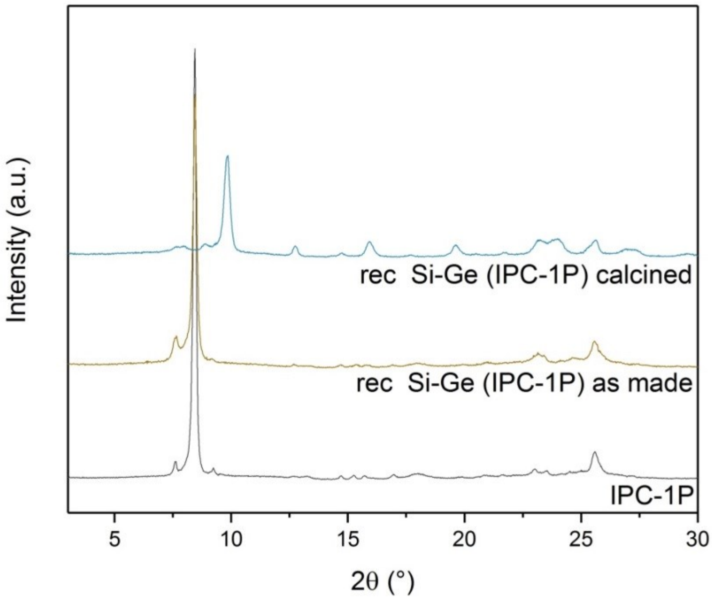

Figure S1: Powder XRD patterns of the parent IPC-1P sample (bottom), the sample treated with a mixture of $(EtO)_2Me_2Si$ and $(MeO)_4Ge$ (middle) and the calcined sample (top).

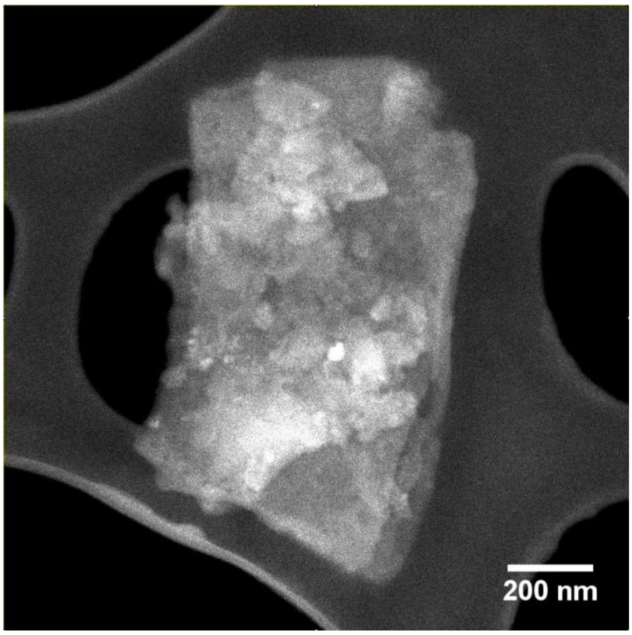

Figure S2: STEM image of the rec Ge sample

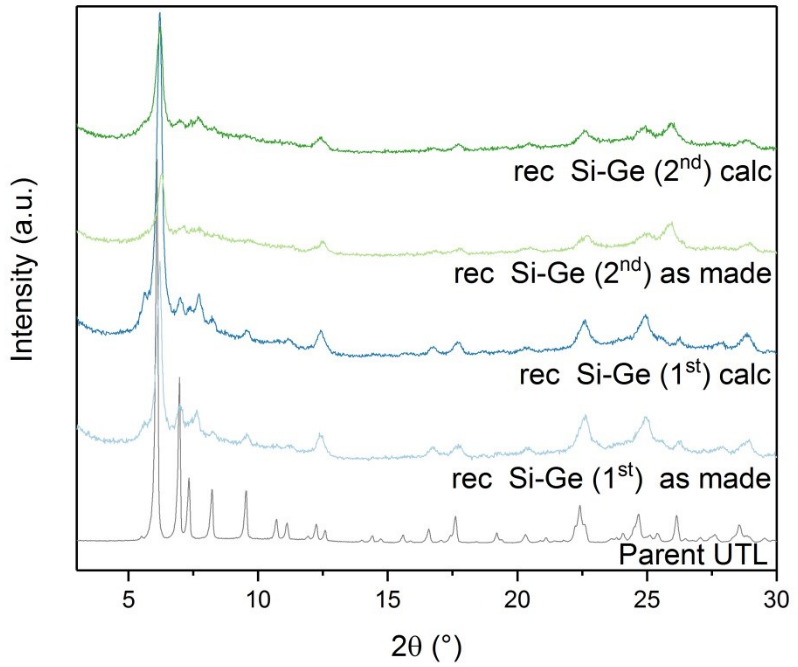

Figure S3: Powder XRD patterns of samples after the 1st and 2nd reconstruction cycle

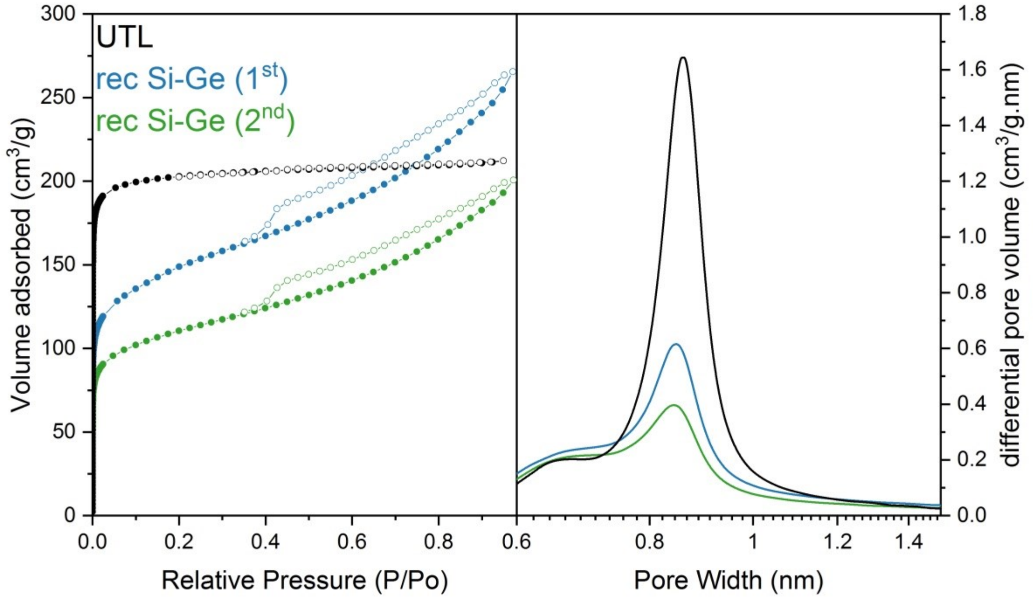

Figure S4: Argon adsorption-desorption isotherms (left) and H-K micropore size distributions (right) of samples after the 1st and 2nd reconstruction cycle

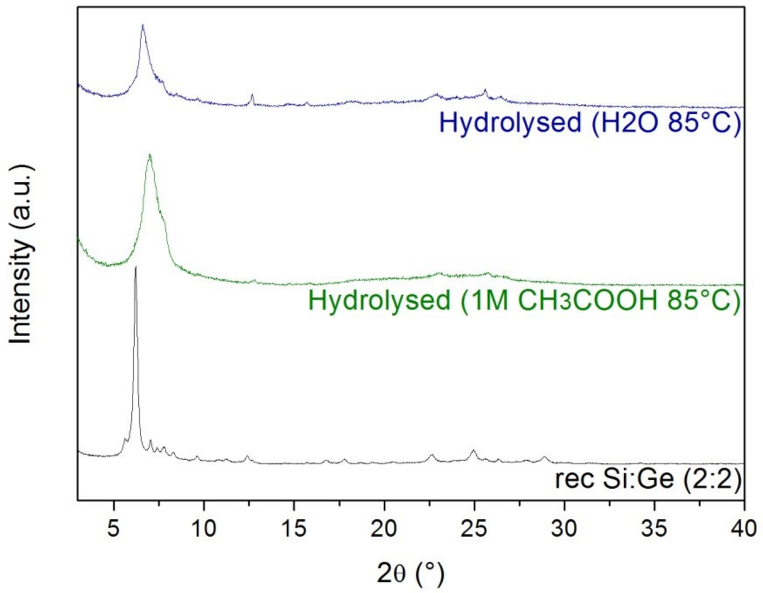

Figure S5: Powder XRD patterns of reconstructed UTL sample before and after hydrolysis in H_2O and acetic acid

Table S1: Textural properties of samples after the 1st and 2nd reconstruction cycle determined by Ar adsorption

	BET (m^2/g)	S_{ext} (m^2/g)	V_{tot} (cm^3/g)	V_{mic} (cm^3/g)
Parent **UTL**	620	52	0.27	0.25
1st run	456	164	0.34	0.10
2nd run	335	127	0.26	0.08

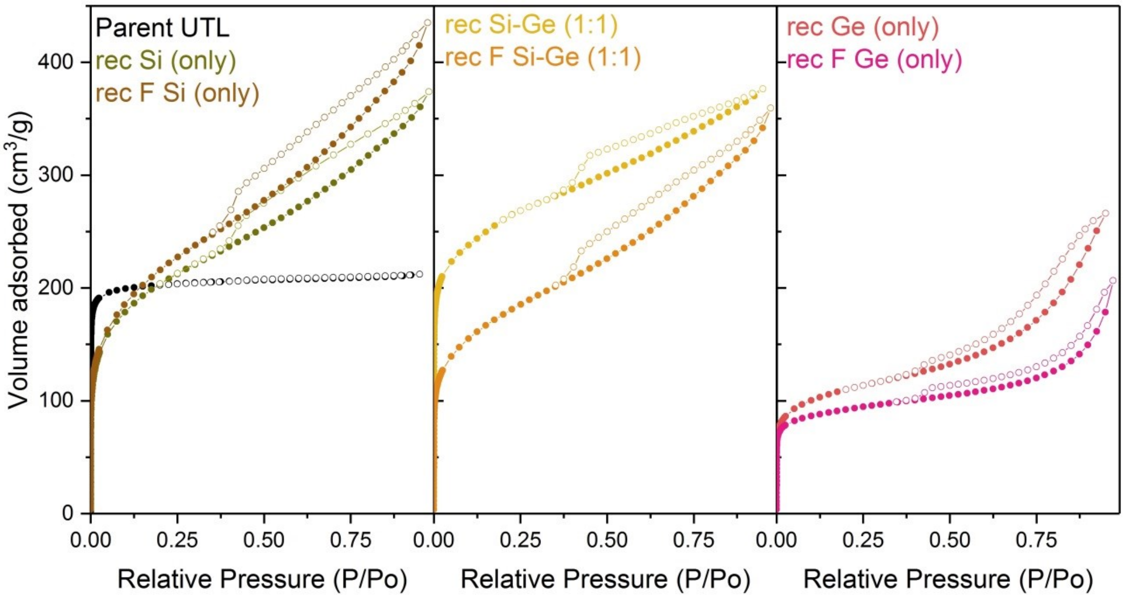

Figure S6: Argon adsorption-desorption isotherms of samples after reconstruction

with different Si:Ge compositions, with and without NH_4F

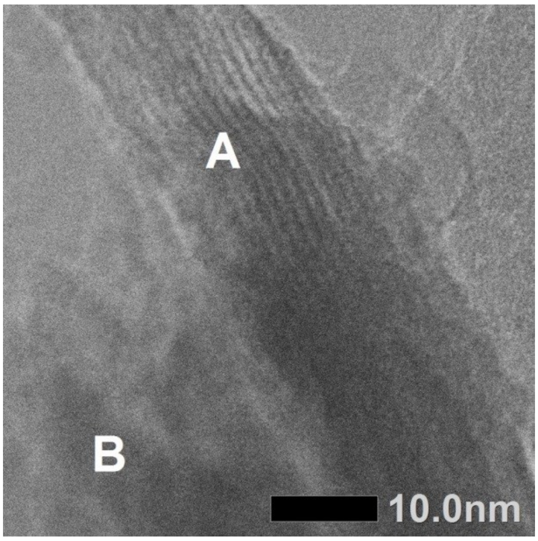

Figure S7: STEM image of the sample rec Si-Ge (1:1) reconstructed in presence of NH_4F, showing both crystalline (A) and amorphous (B) phases

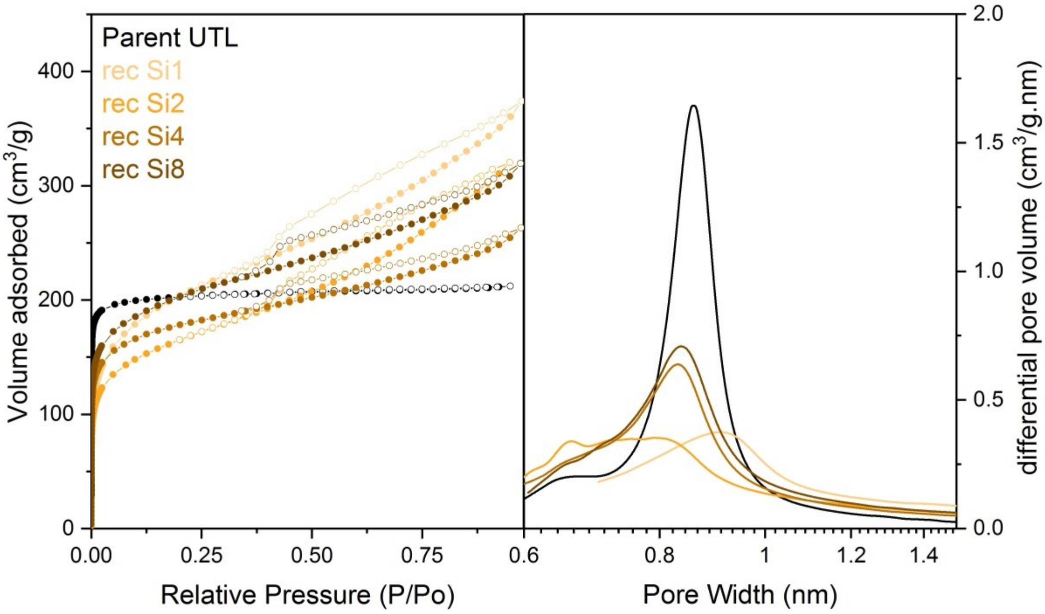

Figure S8: Argon adsorption-desorption isotherms (left) and H-K micropore size distributions (right) of the samples after D4R reconstruction with different Si sources

Table S2: Textural properties of the samples after D4R reconstruction with different Si sources determined by Ar adsorption

	BET (m^2/g)	S_{ext} (m^2/g)	V_{tot} (cm^3/g)	V_{mic} (cm^3/g)
**UTL**	620	52	0.27	0.25
Si1	645	334	0.48	0.12

Si2	542	172	0.34	0.09
Si4	555	131	0.34	0.14
Si8	633	188	0.41	0.13
